# A Case of Hemorrhagic Myositis Associated With Prophylactic Heparin Use in Dermatomyositis

**DOI:** 10.7759/cureus.39540

**Published:** 2023-05-26

**Authors:** Mery Bartl, Jose G Gomez Casanovas, Christine E Loftis, Laura Rincon-Rueda, Andres R Suarez Parraga

**Affiliations:** 1 Internal Medicine, University of Texas Rio Grande Valley School of Medicine, Edinburg, USA; 2 Internal Medicine, University of Texas Rio Grande Valley School of Medicine, McAllen, USA

**Keywords:** intramuscular hemorrhage, muscle hemorrhage, paraneoplastic dermatomyositis, dermatomyositis, anticoagulant therapy, muscle hematoma, paraneoplastic myositis, spontaneous hemorrhage

## Abstract

Dermatomyositis (DM) is a rare systemic autoimmune disease that is associated with inflammation of the skin and muscles. It typically presents with weakness of the proximal muscles along with characteristic skin lesions such as Gottron's papules and heliotrope rash. One of the most feared complications of this disease is the appearance of spontaneous hemorrhagic myositis, as most reported cases are fatal. The mechanism or risk factors of this condition have not been elucidated; however, prophylactic anticoagulation has been correlated with it in previous case reports, although idiopathic hemorrhagic myositis may also be present. We present a case of spontaneous intramuscular hemorrhage (SIH) in a recently diagnosed DM patient. A 59-year-old Hispanic male with a medical history of recently diagnosed prostate cancer and DM presented to the emergency department (ED) due to worsening anemia. His previous hemoglobin (Hgb) was 9 g/dl, but repeated laboratory tests revealed a level of 6.5 g/dl and later 5.5 g/dl at the ED. On admission, the patient was afebrile, tachycardic, and normotensive, with no overt sign of gastrointestinal bleeding. The physical exam revealed an ecchymosis on the right medial aspect of the thigh, and a digital rectal exam was negative. Computer tomography (CT) of the abdomen and pelvis without contrast was ordered due to suspicion of a retroperitoneal hematoma, revealing an interval development of a right groin complex fluid collection of up to 6 cm, concerning a possible hematoma. The patient did not have any previous vascular procedures in the area but was exposed to deep vein thrombosis (DVT) prophylaxis during the previous admission. Vascular surgery was consulted, and the recommendation was made to proceed with conservative management. On the third day, the patient developed new-onset, left-sided pleuritic chest pain. Upon examination, significant swelling and tenderness were noted in his left pectoral region, which was not present on admission. A CT chest without contrast was ordered due to concerns of underlying hematomas, revealing bilateral thickening of the pectoralis muscles, more on the right side, with a fluid collection of 2.5 cm × 1.3 cm. In addition, there was thickening of the right lateral chest wall muscles in the posterior right trapezius or supraspinatus muscles, most likely from intramuscular hemorrhage. The patient was transferred to the step-down unit for close monitoring. Conservative management was continued with as-needed transfusions for three days until hemoglobin stabilized at 9.8 mg/dL. Once stable, the patient was resumed on steroids and immunosuppressive therapy with posterior resolution of the SIH. SIH has been reported in DM, particularly more prominent in those with anti-MDA-5 antibodies. A case series and literature review showed 60.9% mortality within six months in those presenting with SIH, with a poorer prognosis (80% mortality) in those with deep muscle bleeding versus superficial (25%). There is currently no consensus on the treatment approach, and arterial embolization has not been proven effective. In our patient, conservative management with close surveillance and frequent transfusions helped achieve hemodynamic stability. Clinicians should be more aware of these rare but life-threatening complications in patients presenting with DM.

## Introduction

Dermatomyositis (DM) is an autoimmune disease with humoral-mediated systemic involvement. Antigen-specific antibodies are deposited in the microvasculature either through immune complex deposition or by specific anti-endothelial cell binding [1]. This disorder is relatively rare, with various subtypes affecting both children and adults. A characteristic and almost pathognomonic feature is the presence of Gottron’s papules and heliotrope rash. Other skin manifestations, such as malar erythema, photosensitive poikiloderma, violaceous erythema on extensor surfaces, and periungual and cuticular changes, accompany symmetrical muscle proximal weakness. Creatinine kinase (CK) levels are often elevated in patients with DM, and myositis-specific antibodies (MSA) are detectable in approximately 60% of patients. Common MSAs include MDA-5 (anti-melanoma differentiation-associated gene 5), NXP-2 (anti-nuclear matrix protein 2), Mi-2, TIF1-γ (transcription intermediary factor 1-gamma), and SAE-1 (small ubiquitin-like modifier activating enzyme). Spontaneous intramuscular hemorrhage (SIH) is an exceedingly rare but feared complication of DM, with most reported cases being fatal. While the mechanism and risk factors of this entity have not been elucidated, it is generally associated with acute-phase presentation and exposure to prophylactic anticoagulation, although idiopathic cases have also been reported [2,3]. Here, we present a case of SIH in a patient recently diagnosed with DM who was on deep vein thrombosis (DVT) prophylaxis.

## Case presentation

A 59-year-old Hispanic male with a medical history of hypertension, diabetes mellitus type 2, and hyperlipidemia, recently diagnosed with prostate cancer and paraneoplastic DM, with an MSA panel negative on a previous admission, was transferred to the emergency department (ED) from inpatient rehabilitation due to worsening anemia. Prior to being discharged, the patient’s hemoglobin (hgb) was stable at 9 g/dl. However, repeated laboratory studies at the outside facility revealed a significant drop to 6.5 g/dl, which was found to be 5.5 g/dl upon arrival at the emergency department.

**Figure 1:**
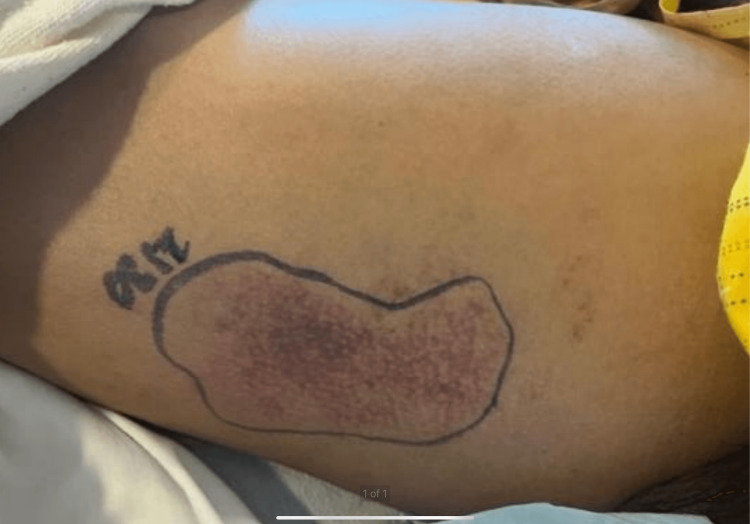
Ecchymosis on the medial portion of the right thigh, present on admission to the hospital.

On admission, the patient’s vital signs were a temperature of 97.7 °F, a heart rate of 110 bpm, a respiratory rate of 20 rpm, a SpO_2_ of 96% in room air, and a blood pressure of 137/74 mm Hg. The patient’s EKG was consistent with sinus tachycardia without acute ST or T wave changes. The physical examination was remarkable for an area of ecchymosis in the medial aspect of the right leg (Figure 1), associated with palpable bilateral femoral and dorsalis pedis pulses, and a digital rectal exam was negative for gross blood. The patient was also found to have anasarca, proximal weakness 3/5 on bilateral lower extremities, an erythematous rash affecting the face and arms diffusely, and Gottron's papules on bilateral hands symmetrically. 

Given the acuity of the drop in Hgb level, an upper gastrointestinal bleed versus intra-abdominal bleeding was suspected. Gastroenterology (GI) was consulted; however, due to the patient's lack of overt signs of bleeding and his unremarkable digital rectal exam, his anemia was deemed to be less likely from a GI source, considering the above information.

**Figure 2:**
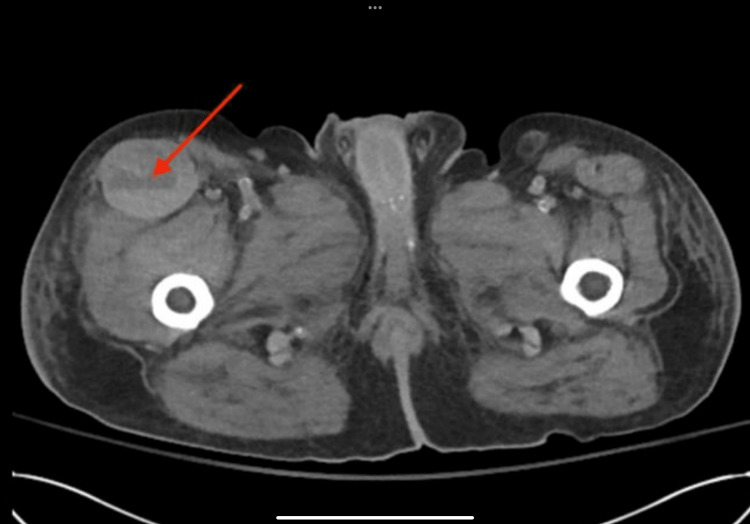
A pelvic CT scan without contrast detected a sizable fluid collection in the right groin to medial aspect of the thigh, suggesting hemorrhagic myositis in a patient recently diagnosed with dermatomyositis.

The Hgb levels were monitored, and since they were found to be less than 7 g/dl, two units of packed red blood cells (pRBCs) were given. After the transfusion, the repeat Hgb test showed an improvement to 7.8 g/dl. CT of the abdomen and pelvis without contrast was ordered to rule out retro-peritoneal hematoma, among other differentials, which revealed an interval development of a right groin complex fluid collection of up to 6 cm concerning a possible hematoma (Figure 2).

**Figure 3:**
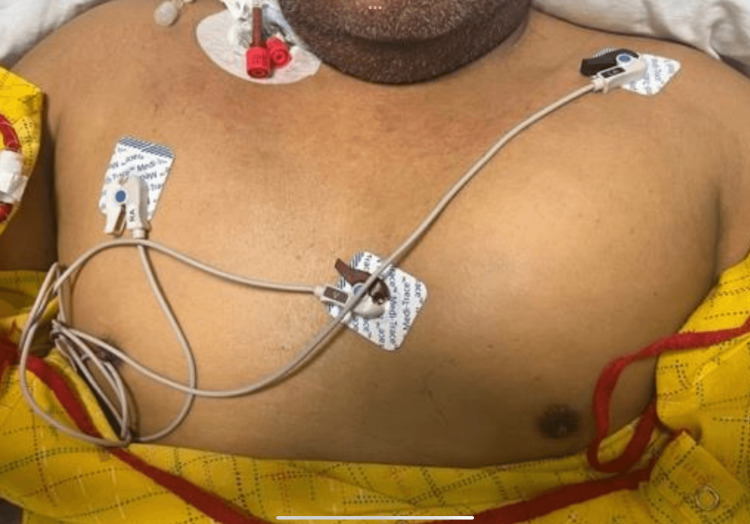
Chest inspection revealing asymmetric swollen left pectoralis area, in a patient with dermatomyositis and hemorrhagic myositis of the left pectoralis muscle.

The patient had no previous vascular procedures in the area or recent trauma yet; he was exposed to chemoprophylaxis for DVT with heparin during the previous admission. To further investigate the fluid collection and rule out pseudoaneurysms, a non-vascular ultrasound (US) was ordered and revealed a complex fluid collection of 11.8 cm × 2.3 cm × 4.8 cm. Vascular surgery was consulted; however, they recommended conservative management, along with serial trending of Hgb levels, as an invasive procedure had no apparent benefit. The next day, the patient continued to require pRBC transfusions, and the patient had a new onset of pleuritic left-sided chest pain. Upon examination, there was significant swelling and tenderness in his left pectoral region, which was not present on admission, and the chest X-ray was unrevealing (Figure 3).

**Figure 4:**
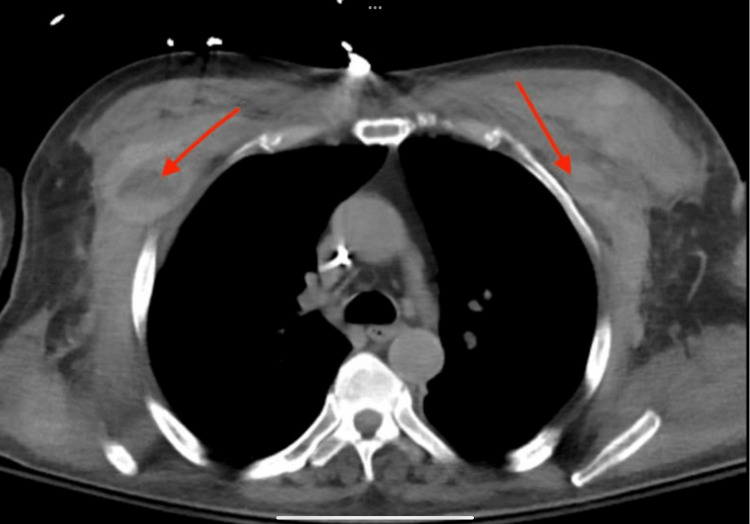
A non-contrast CT scan of the chest revealing bilateral thickening of the pectoralis muscles, with more pronounced involvement on the right side and a fluid collection measuring 2.5 cm × 1.3 cm.

A panel was conducted to evaluate the coagulation profile, which indicated that the international normalized ratio (INR), prothrombin time (PT), and partial thromboplastin time (PTT) were within normal ranges. Additionally, the tests for factor V Leiden and anti-thrombin III were negative. To assess for possible intrathoracic bleeding or intramuscular hemorrhage, a CT chest scan was performed without contrast, which showed bilateral thickening of the pectoralis muscles with more noticeable thickening on the right side and a fluid collection measuring 2.5 cm × 1.3 cm (Figure 4). Additionally, there was thickening in the posterior right trapezius/supraspinatus muscles of the right lateral chest wall, which was most likely indicative of hematomas.

After being given three additional units of pRBC due to refractory anemia (Hgb level 6.1 mg/dL) and concern for impending shock, the patient was transferred to the step-down unit for closer monitoring. At the unit, the patient was managed conservatively and received more transfusions for three days until the Hgb level stabilized at 9.8 mg/dL. No surgical or invasive interventions were deemed necessary. Once the patient's condition stabilized, methylprednisolone was resumed, with a subsequent transition to prednisone orally. Immunosuppressive therapy with mycophenolate mofetil was held due to the patient's hospital course being complicated by MRSA bacteremia, requiring prolonged antibiotic therapy. Also, the patient had a new complaint of oropharyngeal dysphagia with solids and liquids, with frank aspiration on a modified barium swallow study on any food consistency. Esophagogastroduodenoscopy (EGD) was unrevealing, and rheumatology was consulted, with a recommendation made to start the patient on IV immunoglobulin (IVIG) at 2 g/kg per day for two consecutive days without major resolution in the short term. The patient was then transitioned to oral prednisone with a taper. After the resolution of SIH and stabilization of the anemia, the patient underwent a percutaneous endoscopic gastrostomy (PEG) to continue nutrition support, and immunosuppressive therapy with mycophenolate mofetil was re-initiated.

## Discussion

SIH is a rare and neglected complication of DM with high mortality rates of approximately 60%. The incidence and prevalence of this well-described complication of DM are unknown. It predominantly affects adults over 50 years old with no gender preference [4]. The pathophysiology and muscular biopsy findings suggest that intramuscular hemorrhage occurs due to microvascular injury in the setting of myositis. A study demonstrated that muscle biopsies of DM patients with SIH have minimal structural changes, focal capillary depletion of about 50%, and a significant reduction in capillary density [4]. Immune complex formation in the walls of intramuscular venules and arterioles also contributes to microvascular injury [5]. This supports the distinction between non-DM SIH and DM-associated hematomas, as the latter result from diffuse bleeding from multiple small vessels, whereas the former originates from a single artery [3]. Despite normal anticoagulation studies, prophylactic anticoagulation and the use of antiplatelet therapy have been associated with an increased risk of bleeding in DM [2], which may have contributed to or worsened the rapid evolution of pectoral hematoma in our case, given his exposure to prophylactic anticoagulation during his previous admission.

We performed a literature review using the PubMed database and MyUTRGV library to search for studies describing SIH in the context of DM, newly or previously diagnosed. The search was conducted using "SIH," "DM," "hemorrhage," and "hemorrhagic dermato-myositis." The search was performed on January 2023. Our review included 18 patients whose cases were summarized according to clinical and laboratory features as well as outcomes (Table 1) [1-3,6-17]. The patients were predominantly female (55%, n=10), with a median age of 50 at admission. Muscle involvement in SIH is divided by location and depth (deep and superficial muscles). The most frequently involved bleeding location was the iliopsoas, with the psoas-iliac muscle group [1,3,5,6,8-11,17] accounting for 50% (n=9) of the cases, followed by the rectus sheath [6,8,14], obliques abdominis [3,6,14], and limb-girdle muscles [2,7,12,13], with some overlapping on the location among patients. Interestingly, psoas-iliacus is not the most common muscle group described in DM; limb-girdle muscles are the most common one [4]. Other locations were less frequently mentioned, including the pectoralis muscle, as presented in our patient, as well as the paravertebral muscle, latissimus dorsi, bicipital and tricipital muscles, deltoid, trapezius, brachial, and sternocleidomastoid muscles. Greater survival is associated with superficial muscle involvement, which is suspected secondary to earlier recognition, detection, and intervention compared to deep muscle hematoma [8]. Our literature review confirmed these findings, indicating that 73% (n=10) of patients experienced recovery, with 80% (n=8) demonstrating only superficial muscle involvement. In contrast, over 50% (n=6) of patients who passed away had deep muscle involvement. Among these patients with deep muscle SIH, only two were diagnosed with malignancies: hepatocellular carcinoma [7] and ovarian carcinoma with abdominal carcinomatosis [5], representing just 11%. Although DM can sometimes manifest as a paraneoplastic syndrome, our review only identified two such cases, both of which resulted in recovery.

**Table 1:** Summary of reported cases with spontaneous intramuscular hemorrhage related to dermatomyositis. N/A: Not applicable or not reported in the article; anti-MDA5: anti-melanoma differentiation-associated gene 5; anti-NXP-2: anti-nuclear matrix protein 2.

Author	Age	Gender	Location	Underlying malignancy	Platelet count	Coagulation function	Myositis-specific antibodies	Anti-coagulation/anti-platelet therapy	Steroidal therapy	Treatment	Outcome	Reference
Higashi et al. [1]	77	F	Right sternomastoid, left iliopsoas and iliac muscles	No malignancy	Normal	Normal	Anti-KL-6	Heparin and aspirin	Methylprednisolone IV	N/A	Deceased	[1]
Chandler et al. [2]	64	F	Right breast, left thigh	No malignancy	N/A	Prolonged PTT	Anti-Mi-2	Heparin	Methylprednisolone IV	Hematoma evacuation	Recovered	[2]
Xu et al. [3]	41	M	Right psoas	No malignancy	Normal	Normal	Anti-MDA5, Anti-Ro-52	Aspirin	Methylprednisolone IV	Coil embolization	Deceased	[3]
Xu et al. [3]	66	F	Right musculi obliquus Internus abdomini muscle	No malignancy	Normal	Normal	Anti-MDA5, Anti-Ro-52	N/A	Methylprednisolone IV	Conservative	Recovered	[3]
Li Fraine et al. [5]	53	F	Left iliopsoas muscle	Ovarian cancer with abdominal carcinomatosis	Normal	N/A	N/A	Dalteparin	Methylprednisolone IV	Conservative	Hospice	[5]
Orrell et al. [6]	50	F	Left rectus abdominis muscle, left internal, external oblique and latissimus dorsi muscles	No malignancy	Normal	Normal	N/A	N/A	Prednisone	Conservative	Recovered	[6]
Orrell et al. [6]	11	F	Retroperitoneal hematoma	No malignancy	N/A	N/A	N/A	N/A	Prednisolone	Conservative	Recovered	[6]
Suárez-Díaz et al. [7]	77	M	Tricipital and bicipital muscles	Liver cancer	Normal	Normal	N/A	Aspirin and Low molecular weight heparin	Methylprednisolone IV	Conservative	Recovered	[7]
Yamagishi et al. [8]	64	F	Right psoas, right iliacus muscles, left rectus sheath	No malignancy	Normal	Normal	Anti-KL-6	Dalteparin	Methylprednisolone IV	Embolization	Deceased	[8]
Watanabe et al. [9]	82	M	Left iliopsoas	No malignancy	Normal	Normal	Anti-MDA5	N/A	Methylprednisolone IV	None	Deceased	[9]
Chen et al. [10]	60	F	Left psoas	No malignancy	N/A	N/A	N/A	Enoxaparin	Methylprednisolone IV	Embolization	Deceased	[10]
Miwa et al. [11]	65	F	Bilateral iliopsoas muscles and thigh muscles	No malignancy	Decreased	Prolonged PTT	N/A	Unfractionated heparin	Methylprednisolone IV	Conservative	Recovered	[11]
Hanawa et al. [12]	60	M	Left deltoid, trapezius muscle	No malignancy	Normal	Prolonged PTT	N/A	Unfractionated heparin	Methylprednisolone IV	Conservative	Deceased	[12]
Kono et al. [13]	24	M	Brachial muscles	No malignancy	N/A	N/A	Anti-MDA5	None	Methylprednisolone IV	Emergency bypass	Recovered	[13]
Langguth et al. [14]	80	M	Right rectus sheath and oblique muscles	No malignancy	Normal	Prolonged PTT	N/A	Unfractionated heparin	Methylprednisolone IV	Conservative	Recovered	[14]
Ju et al. [15]	46	F	Esophageal involvement	No malignancy	N/A	N/A	N/A	N/A	Methylprednisolone IV	Embolization	Deceased	[15]
Brown et al. [16]	35	M	Right Iliacus muscle	No malignancy	N/A	N/A	Anti-Ro-52 and anti-NXP-2	N/A	Prednisolone	Conservative	Recovered	[16]
Lee and Kim [17]	43	M	Left Iliopsoas, Iliacus and retroperitoneum	No malignancy	Decreased	Normal	N/A	Low molecular weight heparin	Methylprednisolone IV	Conservative	Recovered	[17]

The use of MSA has facilitated the process of diagnosing, classifying, and predicting the course of DM. However, in our review, 10 out of the 18 patients did not have their antibody results reported or mentioned in their case, including our patient, leaving only 44% (n=4) of patients with reported antibodies. Detected antibodies included: anti-Mi 2 [2], anti-KL-6 [1], anti-MDA5 [9,13], anti-RO52 plus MDA5 [3], and anti-RO52 plus NXP-2 [16]. These dual-positive anti-MDA5 and anti-Ro52 antibodies have also been correlated with worse prognosis and poor pulmonary outcomes, especially in the older population [16], and interestingly, patients with anti-KL6, anti-MDA5, and anti-RO52 positivity did not survive. However, there is not enough evidence describing the correlation between these antibodies and prognosis in SIH related to DM.

Regarding the therapy course, 15 out of the 18 cases were managed with intravenous methylprednisolone 1 g daily for three days, followed by prednisone 60 mg daily with taper. Treatment strategies included observation, conservative management with blood transfusions, and more invasive procedures such as embolization. Conservative management was the chosen route for most patients, with Hgb trending and as-needed transfusions instead of surgical intervention. Six patients out of the eighteen cases died due to an SIH-related complication (e.g., hemorrhagic shock, DIC, and sepsis), and the majority of patients (61%) recovered, with one patient placed in hospice with an unknown timing of death.

In terms of using anticoagulation as a chemoprophylaxis for DVT, it is well known that DM increases the risk of venous thromboembolism (VTE). Therefore, when not contraindicated, most of these patients are placed on chemoprophylaxis when admitted to the hospital. However, the risks and benefits of prophylactic anti-thrombotic treatment must be carefully balanced in routine clinical practice, as it can potentially exacerbate SIH and increase the risk of further complications and death [18]. About 57% of patients in the deceased group received anticoagulation and/or antiplatelet therapy for VTE prophylaxis. Among the options for chemoprophylaxis used were low-molecular-weight heparin, enoxaparin, and unfractionated heparin. Li Fraine et al. mentioned a case where the patient was placed on therapeutic dalteparin, not for VTE prophylaxis but for tumor-associated ovarian vein thrombosis. Nonetheless, no tendency was found regarding what type of anticoagulation, therapeutic or prophylactic, was safer for preventing bleeding complications; instead, among the seven patients not on anticoagulation at the time of diagnosis, five survived, and unfortunately, two died, with one being positive for an MDA5-positive antibody. Management of SIH primarily aims to control hemorrhage, which may include embolization and transfusions. Out of the 18 cases, coagulation studies were normal in 14 of them. Among the remaining four cases with abnormal coagulation panels, prolonged PTT was the only abnormality observed. Three of these patients recovered, but unfortunately, one patient died.

The prognosis for SIH is typically poor, with a mortality rate of approximately 50% [18]. In the cases reviewed, embolization was attempted in four patients [3,8,10,15], but all of them succumbed to their condition. Despite advancements in the recognition and management of DM, there is currently no guideline or consensus on the treatment approach for this complication. Arterial embolization has been described in some cases as ineffective [19]. In our patient, conservative management with close surveillance and frequent transfusions was the approach that helped achieve hemodynamic stability. As clinicians, we should be aware of these rare but life-threatening complications in patients presenting with DM.

## Conclusions

In conclusion, the management of SIH in patients with DM remains a challenge, with conservative management being the preferred approach for most patients. The use of anticoagulation as a chemoprophylaxis for DVT in these patients requires careful consideration of the risks and benefits, as it can potentially exacerbate SIH and lead to further complications. Future research is needed to investigate the most effective strategies for preventing and managing SIH in patients with DM and to develop evidence-based guidelines for the treatment of this complication. It is crucial for clinicians to be aware of the risk of SIH in patients with DM and to closely monitor patients who are receiving anticoagulation therapy for any signs of bleeding complications. While most of the case reports mention that most patients with SIH recover, they do not provide information on their long-term outcomes or potential complications. Early recognition and prompt management of SIH are essential to improving patient outcomes.
